# Trends in prior antithrombotic medication and risk of in-hospital mortality after spontaneous intracerebral hemorrhage: the J-ICH registry

**DOI:** 10.1038/s41598-024-62717-5

**Published:** 2024-05-25

**Authors:** Hideaki Ueno, Joji Tokugawa, Rikizo Saito, Kazuo Yamashiro, Satoshi Tsutsumi, Munetaka Yamamoto, Yuji Ueno, Makiko Mieno, Takuji Yamamoto, Makoto Hishii, Yukimasa Yasumoto, Chikashi Maruki, Akihide Kondo, Takao Urabe, Nobutaka Hattori, Hajime Arai, Ryota Tanaka, The Juntendo Registry of Spontaneous Intracerebral Hemorrhage Study Group

**Affiliations:** 1 Department of Neurosurgery, Juntendo University Shizuoka Hospital, 1129 Nagaoka, Izunokuni, Shizuoka 410-2295 Japan; 2 Department of Neurosurgery, Juntendo University Nerima Hospital, 3-1-10 Takanodai, Nerima, Tokyo 177-8521 Japan; 3 Department of Neurosurgery, Koshigaya Municipal Hospital, 10-47-1 Higashikoshigaya, Koshigaya, Saitama 343-0023 Japan; 4 Department of Neurology, Juntendo University Urayasu Hospital, 2-1-1 Tomioka, Urayasu, Chiba 279-0021 Japan; 5 Department of Neurosurgery, Juntendo University Urayasu Hospital, 2-1-1 Tomioka, Urayasu, Chiba 279-0021 Japan; 6 Department of Neurosurgery, Juntendo University School of Medicine, 2-1-1, Hongo, Bunkyo, Tokyo 113-8421 Japan; 7 Department of Neurology, University of Yamanashi, 1110, Shimokato, Chuo, Yamanashi 409-3898 Japan; 8 Department of Neurology, Juntendo University School of Medicine, 2-1-1, Hongo, Bunkyo, Tokyo 113-8421 Japan; 9 Department of Medical Informatics, Center for Information, Jichi Medical University, 3311-1, Yakushiji, Shimotsuke, Tochigi 329-0498 Japan; 10 Stroke Center and Division of Neurology, Department of Medicine, Jichi Medical University, 3311-1, Yakushiji, Shimotsuke, Tochigi 329-0498 Japan

**Keywords:** Cardiology, Medical research, Neurology

## Abstract

Spontaneous intracerebral hemorrhage (SICH) remains a devastating form of stroke. Prior use of antiplatelets or warfarin before SICH is associated with poor outcomes, but the effects of direct oral anticoagulants (DOACs) remain unclear. This study aimed to clarify trends in prior antithrombotic use and to assess the associations between prior use of antithrombotics and in-hospital mortality using a multicenter prospective registry in Japan. In total, 1085 patients were analyzed. Prior antithrombotic medication included antiplatelets in 14.2%, oral anticoagulants in 8.1%, and both in 1.8%. Prior warfarin use was significantly associated with in-hospital mortality (odds ratio [OR] 5.50, 95% confidence interval [CI] 1.30–23.26, P < 0.05) compared to no prior antithrombotic use. No such association was evident between prior DOAC use and no prior antithrombotic use (OR 1.34, 95% CI 0.44–4.05, P = 0.606). Concomitant use of antiplatelets and warfarin further increased the in-hospital mortality rate (37.5%) compared to warfarin alone (17.2%), but no such association was found for antiplatelets plus DOACs (8.3%) compared to DOACs alone (11.9%). Prior use of warfarin remains an independent risk factor for in-hospital mortality after SICH in the era of DOACs. Further strategies are warranted to reduce SICH among patients receiving oral anticoagulants and to prevent serious outcomes.

## Introduction

Spontaneous intracerebral hemorrhage (SICH) is a devastating disorder of the central nervous system with high morbidity and mortality^1,2^. In contrast to the development of hyperacute treatments for ischemic stroke, scientifically proven treatments for acute-stage SICH are still lacking. Population-based epidemiological data have shown decreasing SICH incidence in high-income countries and increasing SICH incidence in low- to middle-income countries over the last four decades^3^. However, the Framingham Heart Study showed continued increases in ICH incidence, particularly among patients ≥ 75 years old^4^. The higher incidence of SICH among elderly patients is probably due to the higher prevalence of hypertension, amyloid angiopathy, and the use of antithrombotic (AT) medications^2,4,5^. Data from the Japanese Hisayama study demonstrated that the incidence of ICH declined significantly from the first cohort (1961–1974) to the second cohort (1974–1987), although no such trend was found in the third cohort (1988–2001), and the incidence of ICH among patients ≥ 80 years old increased constantly over time^6^. Japan is a super-aging country, and individuals using AT medications for the primary or secondary prevention of cardiovascular disease and ischemic stroke are increasing. Further, patients prescribed direct oral anticoagulants (DOACs) as an alternative to warfarin have been increasing since 2011, when these DOACs first became available^7,8^. These practical changes in AT medication use may influence bleeding complications such as SICH, but such data remain scarce in Japan. We therefore investigated current trends in prior AT use with SICH and its effects on in-hospital mortality in the DOAC era.

## Methods

### Study design

The Juntendo registry of spontaneous IntraCerebral Hemorrhage (J-ICH registry) is an observational, multicenter, prospective registry of patients with SICH. Patient enrollment ran from September 1, 2016, to December 31, 2019 at 5 stroke centers located in Tokyo or neighboring regions in Japan. We obtained informed consent from all participants and combined this data with a retrospective study of SICH for patients from whom consent could not be obtained due to disease severity and/or a lack of legal representatives, with an opt-out option. We enrolled patients with SICH who were ≥ 20 years old and with onset ≤ 7 days prior, excluding patients with bleeding due to arteriovenous malformations, brain tumors, blood disorders (e.g., coagulopathy, thrombocytopenia, leukemia), severe liver dysfunction, or collagen diseases. We collected clinical information from medical records such as age, sex, body mass index (BMI), vascular risk factors, prior AT medication, stroke severity (National Institutes of Health Stroke Scale, NIHSS), modified Rankin Scale (mRS) and mortality at discharge. We measured hematoma volume (HV) and collected information on intraventricular hemorrhage (IVH), hematoma growth (HG), use of reversal agent (vitamin K2, prothrombin complex concentrate [PCC], fresh frozen plasma [FFP], idarucizumab) and surgical treatment. HV was assessed using the ABC/2 method^9^, and HG was defined as a > 33% or > 6-ml increase relative to baseline hematoma volume within 72 h^10^. We investigated short-term outcomes such as in-hospital mortality and poor outcome (mRS 5-6) at discharge. We then analyzed associations between prior AT use and these outcomes.

### Statistical analysis

Continuous variables were compared using either Student’s *t*-test or the Mann–Whitney U test, and analysis of variance (ANOVA) or the Kruskal–Wallis test were used for comparisons between three or more groups, as appropriate, after testing for the normality of distributions. Frequencies of categorical variables were compared using the χ^2^ test. Multivariate logistic regression analysis was performed to assess the risks of mortality and poor outcomes during hospitalization, adjusting for clinical variables identified by univariate analysis as showing significant or marginally significant (0.05 ≤ P < 0.10) differences between the presence or absence of each outcome. All analyses were performed using JMP version 14.2.0 software (SAS Institute Inc., Cary, NC, USA). Values of P < 0.05 were considered significant.

The ethics review committee at Juntendo University School of Medicine and the relevant ethics committees at all participating sites approved this study. The study was conducted in accordance with the ethical principles of the Declaration of Helsinki and the Ethical Guidelines for Medical and Health Research involving Human Subjects by the Ministry of Health, Labor, and Welfare of Japan.

### Ethical approval

All procedures performed in studies involving human participants were in accordance with the ethical standards of the institutional research committee and with the 1964 Helsinki declaration and its later amendments or comparable ethical standards.

### Informed consent

Informed consent was obtained from all individual participants included in the study. We also combined retrospective data on SICH for patients from whom consent could not be obtained due to disease severity and/or a lack of legal representatives, with an opt-out option.

## Result

### Baseline characteristics and prior antithrombotic medication in SICH

**Figure 1:**
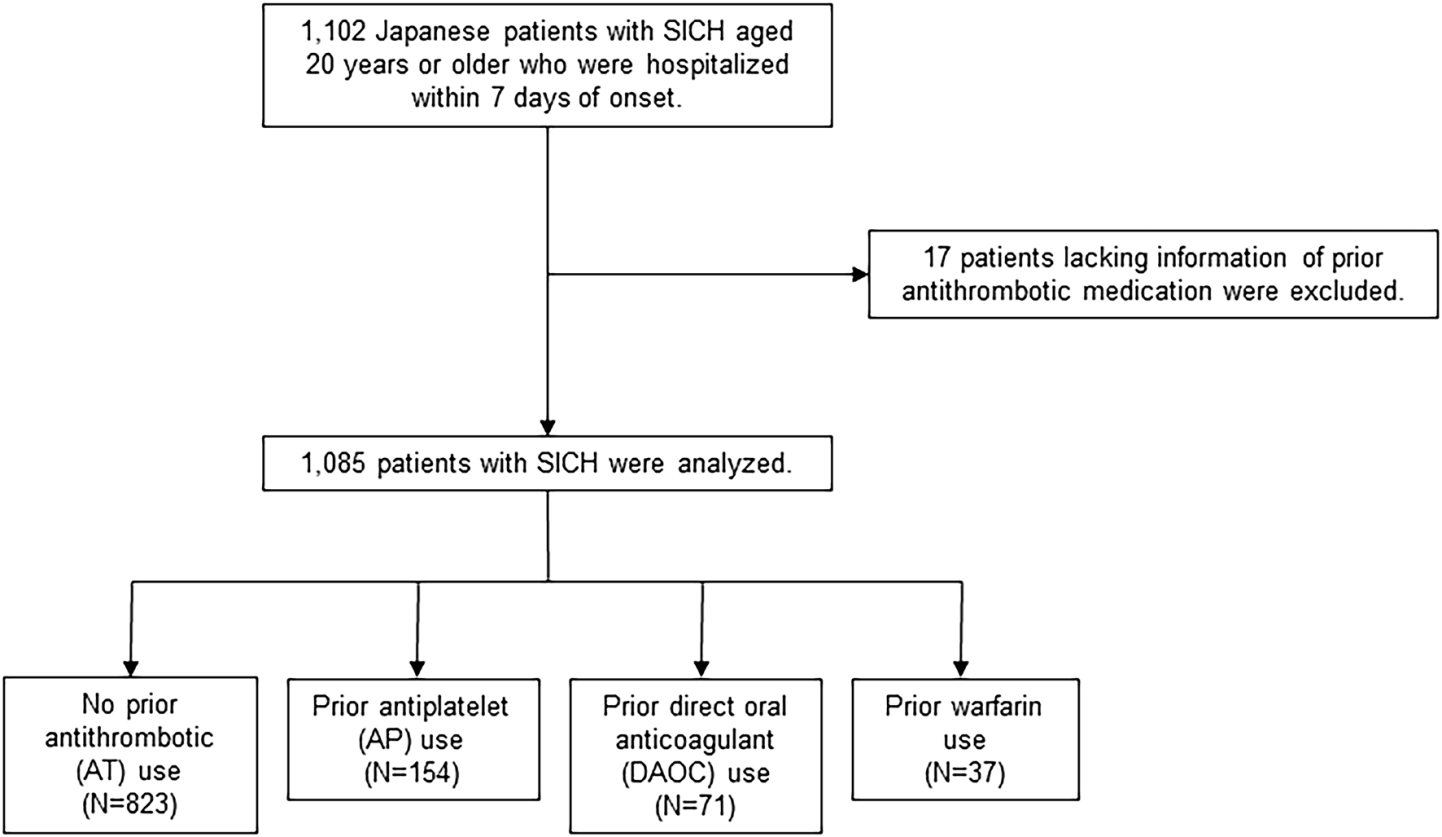
Flowchart of patient selection in the current study.

**Figure 2:**
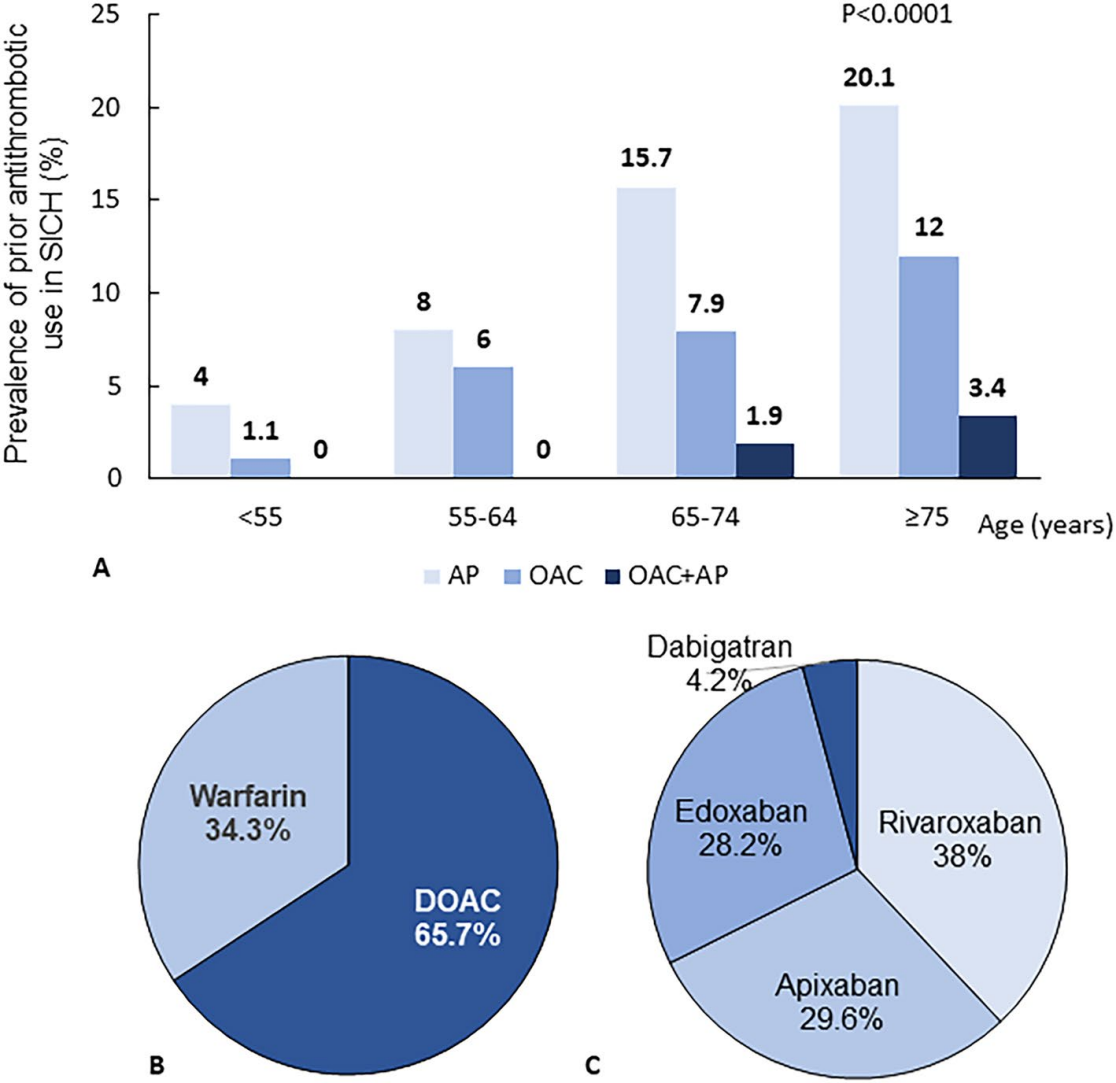
Prior use of antithrombotic (AT) medications in SICH. (**A**) Age-dependent prevalence of each AT medication. (**B**) Prior use of oral anti-coagulant (DOAC vs warfarin). (**C**) Prior use of DOAC subtypes. AP: antiplatelet; DOAC: direct oral anticoagulant; OAC: oral anticoagulant; SICH: spontaneous intracerebral hemorrhage.

A total of 1102 cases of SICH were enrolled. After excluding cases lacking information on prior AT usage, 1085 patients were included for analysis (Fig. 1, Table 1). Among these 1085 enrolled patients, data were obtained retrospectively for only 24 patients (2.2%). Median age (interquartile range: IQR) was 71 years (60–80), and 598 patients (55.1%) were male. Hypertension was the highest vascular risk factor (69.5%), and atrial fibrillation (AF) was present in 8.6%. Previous ischemic stroke was observed in 9.8%, and coronary artery disease (angina pectoris or myocardial infarction) in 5.6%. Median time (IQR) from onset to arrival was 2 h (1–5), and median NIHSS score (IQR) on arrival was 10 (4–18). AT use before SICH was found in 262 patients (24.1%), comprising antiplatelet (AP) use in 154 (14.2%), oral anticoagulant (OAC) use in 88 (8.1%), and use of both AP and OAC (AP + OAC) in 20 (1.8%). The frequency of each prior AT use increased in an age-dependent manner and 35.5% of patients ≥ 75 years old had received some form of AT (Fig. 1). Among patients receiving AP before SICH, aspirin (58.6%) was the most frequent, and dual AP therapy (DAPT) was used in 26 (14.9%) (Supplemental Table 1). Among those receiving OACs, DOACs (65.7%) were used almost twice as often as warfarin (34.3%) (Fig. 2). Among patients receiving DOACs, dabigatran was the least used (4.2%), followed by edoxaban (28.2%), apixaban (29.6%), and rivaroxaban (38.0%), respectively (Fig. 2).

**Table 1:** Baseline characteristics and clinical outcomes according to prior antithrombotic medication.

	Total	No ATT	AP	DOAC	Warfarin	P value
Patients, n	1085	823	154	71	37	
Age, median (IQR), year	71 (60–80)	68 (58–78)	78 (70–83)***	77 (71–83)***	76 (68–83.5)**	< 0.0001
Sex (male), n (%)	598 (55.1%)	440 (53.5%)	89 (57.8%)	48 (67.6%)	21 (56.8%)	0.118
BMI, median (IQR), kg/m^2^	22.4 (20.0–25.2)	22.5 (20.0–25.4)	22.0 (19.4–24.6)	22.6 (20.4–24.7)	21.6 (18.9–24.6)	0.186
Pre-stroke mRS ≥ 3, n (%)	68 (6.3%)	39 (4.7%)	21 (13.6%)	5 (7.0%)	3 (8.1%)	< 0.001
Current smoker, n (%)	203 (18.7%)	178 (21.6%)	14 (9.1%)	9 (12.7%)	2 (5.4%)	< 0.001
Regular drinker, n (%)	283 (26.1%)	234 (28.4%)	19 (12.3%)	23 (32.4%)	7 (18.9%)	< 0.001
Vascular risks and medical history, n (%)						
Hypertension	754 (69.5%)	561 (68.2%)	115 (74.7%)	48 (67.6%)	30 (81.1%)	0.164
Dyslipidemia	380 (35.0%)	256 (31.1%)	81 (52.6%)	26 (36.6%)	17 (46.0%)	< 0.0001
Diabetes mellitus	200 (18.4%)	122 (14.8%)	52 (33.8%)	17 (23.9%)	9 (24.3%)	< 0.0001
Atrial fibrillation	93 (8.6%)	3 (0.4%)	9 (5.8%)	58 (81.7%)	23 (62.2%)	< 0.0001
Hemodialysis	41 (3.8%)	18 (2.2%)	21 (13.6%)	0	2 (5.4%)	< 0.0001
Coronary artery disease	61 (5.6%)	8 (1.0%)	34 (22.1%)	12 (16.9%)	7 (18.9%)	< 0.0001
Ischemic stroke	106 (9.8%)	23 (2.8%)	61 (39.6%)	11 (15.5%)	11 (29.7%)	< 0.0001
Hemorrhagic stroke	106 (9.8%)	74 (9.0%)	20 (13.0%)	9 (12.7%)	3 (8.1%)	0.366
Acute status						
SBP on arrival, median (IQR), mmHg	175 (156–196)	177 (158–199)	172 (157–190)	168 (147–184)*	164 (146–189)*	< 0.001
NIHSS on arrival, median (IQR)	10 (4–18)	9 (4–17)	10 (4–20)	12 (4–19)	10 (6–27)	0.164
Hematoma location and characteristics						
Supratentorial deep	674 (62.3%)	525 (64.0%)	86 (55.8%)	40 (56.3%)	23 (62.2%)	0.187
Lobes	254 (23.5%)	187 (22.8%)	38 (24.7%)	23 (32.4%)	6 (16.2%)	
Cerebellum/brainstem	143 (13.2%)	100 (12.2%)	27 (17.5%)	8 (11.3%)	8 (21.6%)	
Other	12 (1.1%)	9 (1.1%)	3 (2.0%)	0	0	
Hematoma volume, median (IQR), ml	13.5 (5–30)	12.4 (5–30)	14 (6–35.5)	18 (7.2–45)	15 (4.5–56.5)	0.055
Intraventricular hemorrhage, n (%)	393 (36.2%)	274 (33.3%)	74 (48.1%)	28 (39.4%)	17 (46.0%)	0.002
Hematoma growth, n (%)	45 (4.1%)	27 (3.3%)	4 (2.6%)	8 (11.3%)	6 (16.2%)	< 0.0001
Treatment						
Any surgery, n (%)	276 (25.4%)	205 (24.9%)	43 (27.9%)	18 (25.4%)	10 (27.0%)	0.879
Reversal agent use, n (%)	13 (1.2%)	0	0	4 (5.6%)	9 (24.3%)	< 0.0001
Outcomes, n (%)						
In-hospital mortality	80 (7.4%)	43 (5.2%)	21 (13.6%)	8 (11.3%)	8 (21.6%)	< 0.0001
Hospital death within 24 h	25 (2.3%)	12 (1.5%)	9 (5.8%)	1 (1.4%)	3 (8.1%)	< 0.001
Poor outcome (mRS 5–6 at discharge)	201 (18.5%)	121 (14.7%)	49 (31.8%)	16 (22.5%)	15 (40.5%)	< 0.0001

***P < 0.0001, **P < 0.01, *P < 0.05.

### Characteristics of hematoma and acute treatment

Median HV (IQR) was 13.5 ml (5–30), IVH was found in 393 (36.2%), and HG within 72 h was found in 45 (4.1%) (Table 1). The most prevalent hematoma location was the supratentorial deep (62.3%), followed by the lobes (23.5%), and cerebellum/brainstem (13.2%) (Table 1). When more details of hematoma location were categorized into age strata (< 55, 55–64, 65–74, or ≥ 75 years old), the putamen was the most frequent for patients < 55 years old, while the thalamus and lobar locations were the most frequent for those ≥ 75 years old (Supplemental Table 2). Hematoma evacuation was performed in 144 (13.2%), followed by endoscopy-guided aspiration in 97 (8.9%), and external ventricular drainage in 70 (6.5%). A reversal agent (prothrombin complex concentrate (PCC), fresh frozen plasma (FFP), idarucizumab) was used in 13 patients (12.0%) with prior OAC. Details of the reversal agents used are described in Supplemental Table 3.

### Comparison of clinical and outcome features with or without prior AT use

We compared baseline characteristics, status of hematoma, and outcomes by each type of prior AT: no prior antithrombotic therapy (no ATT, n = 823); prior use of AP (n = 154); DOAC (n = 71); or warfarin (n = 37), respectively (Table 1). Patients with prior AT use were older and showed a higher prevalence of vascular risk factors and histories of ischemic stroke and coronary artery disease. Atrial fibrillation (AF) was significantly more frequent among patients with DOACs (81.7%) and warfarin (62.2%). Median HV tended to be greater in patients receiving AT, but the difference was not significant (P = 0.055). HG was highest in patients with warfarin (16.2%), followed by those with DOACs (11.3%), no ATT (3.3%), and AP (2.6%, P < 0.0001). Details of differences in hematoma location by each AT are also shown in Supplemental Table 2. The prevalence of lobar hemorrhage was highest with DOACs (32.4%), and the prevalence of cerebellar hemorrhage was highest with warfarin (13.5%). In-hospital mortality was highest in patients with warfarin (21.6%) followed by AP (13.6%), DOACs (11.3%), and no ATT (5.2%, P < 0.0001). A similar trend was observed in hospital deaths within 24 h (8.1% vs 5.8% vs 1.4% vs 1.5%, P < 0.001) and poor outcome (modified Rankin Scale (mRS) 5–6) at discharge (40.5% vs 31.8% vs 22.5% vs 14.7%, P < 0.0001), respectively. No significant differences were identified in clinical background, hematoma location, hematoma characteristics, and outcomes by each type of DOAC (Supplemental Table 4).

### Risk of in-hospital mortality and poor outcome by prior antithrombotic use

We compared the risk of in-hospital mortality and poor outcome in patients with each prior use of AT compared to no ATT (Table 2). The risk of in-hospital mortality was significant for all types of AT compared to no ATT (odds ratio [OR] for AP 2.86, 95% confidence interval [CI] 1.64–4.98, OR for DOAC 2.30, 95% CI 1.04–5.11; OR for warfarin 5.00, 95% CI 2.16–11.6). After adjusting for covariate (Supplemental Table 5), including pre-stroke mRS, surgical treatment and use of reversal agents, only warfarin remained significantly associated with in-hospital mortality (adjusted OR [aOR] 5.50, 95% CI 1.30–23.26, P < 0.05) compared to no ATT. A similar trend was observed for hospital deaths within 24 h and poor outcomes at discharge, but the significance of these findings disappeared after adjusting for confounders.

**Table 2:** Risk of in-hospital mortality, hospital death within 24 h, and poor outcome (mRS 5–6 at discharge) by prior antithrombotic use.

	Univariate			Age & sex adjusted			Multivariate		
	OR	95% CI	P value	OR	95% CI	P value	OR	95% CI	P value
In-hospital mortality									
No ATT	1 (reference)			1 (reference)			1 (reference)		
AP	2.86	1.65–4.98	< 0.001	2.52	1.42–4.47	< 0.01	0.87	0.34–2.19	0.765
DOAC	2.30	1.04–5.11	0.04	1.91	0.84–4.35	0.122	1.34	0.44–4.05	0.606
Warfarin	5.00	2.16–11.6	< 0.001	4.53	1.93–10.61	< 0.001	5.50	1.30–23.26	< 0.05
Hospital death within 24 h									
No ATT	1 (reference)			1 (reference)			1 (reference)		
AP	4.19	1.74–10.14	< 0.01	4.01	1.58–10.15	< 0.01	1.55	0.32–7.41	0.584
DOAC	0.97	0.12–7.53	0.973	0.86	0.11–6.93	0.885	0.58	0.05–6.17	0.651
Warfarin	5.96	1.61–22.12	< 0.01	5.78	1.52–21.96	< 0.05	3.28	0.36–29.73	0.290
mRS 5–6 at discharge									
No ATT	1 (reference)			1 (reference)			1 (reference)		
AP	2.71	1.83–4.00	< 0.0001	2.28	1.53–3.41	< 0.0001	1.44	0.74–2.80	0.278
DOAC	1.69	0.94–3.04	0.081	1.37	0.75–2.52	0.303	1.03	0.44–2.38	0.952
Warfarin	3.96	2.00–7.84	< 0.0001	3.48	1.74–6.95	< 0.001	2.82	0.90–8.77	0.074

In addition to age and sex, multivariate analysis was adjusted 1) by pre-stroke mRS, regular drinking, hypertension, diabetes mellitus, hemodialysis, coronary artery disease (CAD), systolic blood pressure (SBP) on arrival, NIHSS, hematoma volume (HV), intraventricular hemorrhage (IVH), hematoma growth (HG), any surgery, and use of reversal agents for in-hospital mortality, 2) by pre-stroke mRS, regular drinking, hemodialysis, CAD, ischemic stroke (IS), SBP on arrival, NIHSS, HV, IVH, any surgery, and reversal agents use for hospital death within 24 h, and 3) by BMI, pre-stroke mRS, current smoking, regular drinking, dyslipidemia, diabetes mellitus, hemodialysis, CAD, IS, SBP, NIHSS, HV, IVH, HG, any surgery, and use of reversal agents for poor outcome (mRS 5–6 at discharge).

Among the 25 patients with warfarin use and a prothrombin time international normalized ratio (PT-INR) ≥ 2, five patients (20.0%) received prothrombin complex concentrate (PCC), and 1 (4%) received fresh frozen plasma (FFP). No significant differences in HG or in-hospital mortality were seen between patients with and without treatment (33.3% vs 10.5% for HG, 16.7% vs 15.8% for in-hospital mortality, respectively). In patients with DOAC, only 4 patients (5.6%) received a reversal agent (one received idarucizumab, two received PCC, and one received FFP; Supplemental Table 3). Use of a reversal agent was not associated with any change in outcomes (data not shown).

### Comparison of fatal cases within or after 24 h from hospitalization

To determine the impact on mortality of each AT medication after SICH, we compared clinical and hematological characteristics and hematoma location of patients who died within or more than 24 h from hospitalization (Table 3). Among patients who died within 24 h, all cases showed very severe neurological impairment (NIHSS score), and a large HV except in one case with prior DOAC use. Only one patient with prior DOAC use died in this group showed small HV (18 ml), but hematoma was located in brainstem.

**Table 3:** Comparison of fatal cases within or more than 24 h after hospitalization.

	Mortality within 24 h					Mortality more than 24 h after hospitalization				
	No ATT (n = 12)	AP (n = 9)	DOAC (n = 1)	Warfarin (n = 3)		No ATT (n = 31)	AP (n = 12)	DOAC (n = 7)	Warfarin (n = 5)	
Age, median (IQR), y	71.5 (49.3–86.8)	71 (66.5–81)	82	76 (70–79)	0.821	66 (54–84)	84 (80.5–86)	82 (81–92)	80 (67–86.5)	0.021
Sex (male), n (%)	8 (66.7%)	7 (77.8%)	1 (100%)	2 (66.7%)	0.862	16 (51.6%)	10 (83.3%)	3 (42.9%)	3 (60.0%)	0.226
pre-stroke mRS ≥ 3, n (%)	1 (8.3%)	0	0	0	0.770	4 (12.9%)	2 (16.7%)	1 (14.3%)	0	0.822
NIHSS	37 (30.5–40)	40 (28.5–40)	40	40 (36–40)	0.627	34 (13–40)	34.5 (18.5–40)	31 (28–36)	30 (21.5–37)	0.998
Hematoma characters										
Hematoma volume (HV), median (IQR), ml	57 (14.1–126.3)	67.5 (10.2–100.5)	18	70 (64–119.1)	0.788	55 (16–91.8)	42 (25–97.5)	50 (40–145)	55 (25–67)	0.802
Intraventricular hematoma (IVH), (%)	11 (91.7%)	7 (77.8%)	1 (100%)	3 (100%)	0.662	25 (80.7%)	9 (75.0%)	4 (57.1%)	3 (60.0%)	0.516
Hematoma growth (HG), (%)	0	0	0	0	0	5 (16.1%)	1 (8.3%)	2 (28.6%)	3 (60.0%)	0.085
Any surgery	1 (8.3%)	1 (11.1%)	0	0	0.926	13 (41.9%)	5 (41.7%)	0	1 (20.0%)	0.159
Hematoma location										
Supratentorial deep	7 (58.3%)	3 (33.3%)	0	1 (33.3%)	0.539	17 (54.8%)	6 (50.0%)	2 (28.6%)	4 (80.0%)	0.051
Lobes	0	2 (22.2%)	0	0		5 (16.1%)	1 (8.3%)	5 (71.4%)	0	
Cerebellum/Brainstem	5 (41.7%)	3 (33.3%)	1 (100%)	2 (66.7%)		7 (22.6%)	3 (25.0%)	0	1 (20.0%)	
Other	0	1 (11.1%)	0	0		2 (6.5%)	2 (16.7%)	0	0	

Among patients who died more than 24 h after hospitalization, median age was higher in patients with any AT use than in no ATT. Grade of neurological severity and HV were still high, but milder than in cases who died within 24 h. The rate of HG was much higher in case with prior use of warfarin (60%) compared to use of DOACs (28.6%), no ATT (16.1%), and AP (8.3%).

### Comparison of in-hospital mortality and poor outcome with combination of antiplatelet and oral anticoagulant

**Figure 3:**
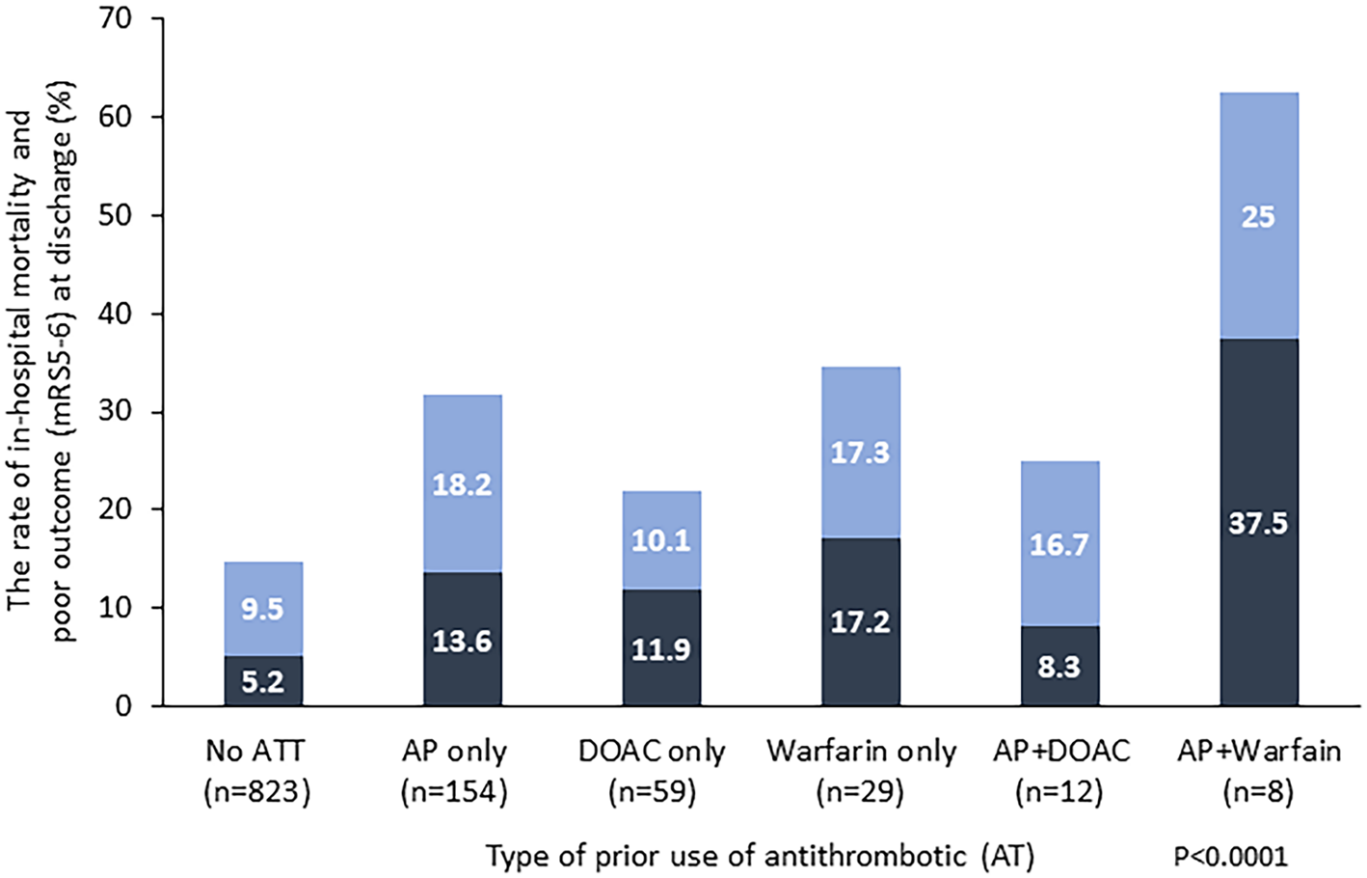
Association between prior antithrombotic medication and in-hospital mortality and functional outcome (mRS5-6) at discharge after SICH, dense bar: mRS6, light bar: mRS5.

Finally, we compared the poor outcome (mRS5-6 at discharge) and in-hospital mortality with or without concomitant use of an antiplatelet among patients receiving an OAC (Fig. 3). Significant differences in rates of in-hospital mortality and poor outcome were evident among each group (P < 0.0001), and the AP + warfarin showed higher rates of in-hospital mortality and poor outcome than no ATT (37.5% vs 5.2%, and 62.5% vs 14.7%, respectively). The rate of in-hospital mortality and poor outcome was higher in AP + Warfarin than warfarin alone (37.5% vs 17.2% for in-hospital mortality, 62.5% vs 34.5 for poor outcome, respectively), but no such trend was observed between AP + DOAC and DOAC alone (8.3% vs 11.9% for in-hospital mortality, 25.0% vs 22.0% for poor outcome, respectively). When comparing several clinical background factors, hematoma location/characteristics, and treatment provided (OAC alone or OAC with concomitant use of AP) (Table 4), we did not find any significant differences in age, sex, rate of pre-stroke mRS ≥ 3, hematoma location/characteristics, or treatment provided in patients receiving DOACs. However, a tendency toward higher HV and a significantly higher rate of IVH were observed in patients receiving AP + warfarin compared to those on warfarin alone. Patients who received any surgery or a reversal agent were also more frequent among patients receiving AP + warfarin than among those on warfarin alone.

**Table 4:** Comparison of baseline, hematoma characteristics, and treatment between monotherapy and dual therapy.

	DOAC only	AP + DOAC	P value	Warfarin only	AP + Warfarin	P value
	(n = 59)	(n = 12)		(n = 29)	(n = 8)	
Age, median (IQR), y	78 (69–84)	77 (75–82.5)	0.503	76 (64.5–83)	78 (73.3–85)	0.17
Sex (male), n (%)	39 (66.1%)	9 (75.0%)	0.548	17 (58.6%)	4 (50.0%)	0.663
Pre-stroke mRS ≥ 3, n (%)	5 (8.5%)	3 (10.3%)	0.295	3 (10.3%)	0	0.343
NIHSS, median (IQR)	12 (4–20)	10 (2.3–18.8)	0.6532	9 (5.5–21)	23 (8.5–39)	0.0840
Laboratory data						
PT-INR	1.18 ± 0.20	1.15 ± 0.20	0.467	2.41 ± 1.04	1.97 ± 0.40	0.178
APTT	34.1 ± 9.5	30.4 ± 4.3	0.245	41.4 ± 12.1	39.0 ± 6.0	0.941
Hematoma characteristics						
Hematoma volume (HV), median (IQR), ml	18 (7.2–45)	18.5 (8–34.5)	0.788	12 (4–40.5)	56.5 (11–77.5)	0.086
Intraventricular hematoma (IVH), (%)	25 (42.4%)	3 (25.0%)	0.262	10 (34.5%)	7 (87.5%)	< 0.01
Hematoma growth (HG), (%)	8 (13.6%)	0	0.176	5 (17.2%)	1 (12.5%)	0.747
Treatment						
Any surgery, (%)	16 (27.1%)	2 (16.7%)	0.448	6 (20.7%)	4 (50.0%)	0.098
Reversal agent use (PCC, FFP, idarucizumab), (%)	4 (6.8%)	0	0.353	5 (17.2%)	4 (50.0%)	0.055
Hematoma location						
Supratentorial deep	32 (54.2%)	7 (58.3%)	0.951	16 (55.2%)	4 (50.0%)	0.374
Lobes	19 (32.2%)	4 (33.3%)		6 (20.7%)	0	
Cerebellum/Brainstem	7 (11.9%)	1 (8.3%)		5 (17.2%)	3 (37.5%)	
Other	1 (1.7%)	0		2 (6.9%)	1 (12.5%)	

## Discussion

This multicenter registered study identified trends in AT use before SICH and revealed prior use of warfarin, but not of AP or DOACs, offered an independent risk factor for in-hospital mortality compared to no prior AT use. Further, patients with concomitant use of warfarin and antiplatelets showed a higher rate of in-hospital mortality and poor outcome than patients with no AT use or other combination AT use.

Before DOACs became available, three Japanese retrospective studies of SICH showed prior AP use in 17.9–20.2%, OAC (warfarin) use in 2.6–6.7%, and AP + OAC use in 1.5–2.8%^11–13^. A larger nationwide population-based cohort study in Taiwan^14^ showed lower prior AP use (12.0%), and OAC use (1.9%) than in Japanese studies. Meanwhile, investigations from Western countries (Italy, The Netherlands, Germany, and the United States) in the same periods^15–18^ showed much higher prevalence for prior use of both AP (25.1–25.6%) and OAC (8.8–25.8%) than East Asia. This may be due to the higher frequency of cardiovascular disease or AF in Western countries than in East Asia^19–21^.

Since DOACs became available, the prevalence of AT use has been changing. Similar to our data, three studies from Japan and Korea have shown much higher use of prior OACs (10.4–13.2%) after DOACs became available^22–24^, while a larger-scale investigation from August 2015 to July 2019 in China still showed a low prevalence of OAC use (1.9%)^25^. Trends in prior use of both AP (24.3–28.2%) and OAC (14.1–21.9%) seemed comparable to data from the United States and Europe between before and after DOACs became available^26–28^.

The incidence of ICH with prior OAC use (OAC-ICH) increased from 0.8 per 100,000 in 1988 to 4.4 per 100,000 in 1999, before DOACs became available in the United States^18^. However, two recent studies from Denmark and the Netherlands showed that the incidence of OAC-ICH was decreasing from before and after DOACs became available (33 or 10.5 per 100,000 person-years to 24 or 7.7 per 100,000 person-years, respectively)^29,30^. These findings may reflect DOACs having about a 50% lower risk of intracranial bleeding compared to warfarin in patients with AF^31^. A key question is whether similar trends will be observed in East Asians, which have about double the incidence of SICH compared to other ethnicities^2^, but no such longitudinal studies have yet been undertaken.

The rate of prior OAC use comprised about two-thirds DOACs in our study. Several previous studies have shown higher rates of warfarin than DOACs among prior users of OACs in SICH^24,26–29^, but a more recent report demonstrated a similar or higher rate of DOAC use compared to warfarin use^32^. Because real-world Japanese data indicated that the use of OACs was increasing and DOACs were rapidly replacing warfarin in patients with non-valvular atrial fibrillation^7^, these situations might result in an increment in the rate of prior DOAC users in Japanese SICH. Indeed, a prospective Japanese multi-stroke center cohort (the PASTA registry) that enrolled acute stroke patients receiving prior OAC from April 2016 to September 2019 showed a similar rate of prior OAC use in SICH (DOACs 73% vs warfarin 27%)^33^.

Although prior AP or warfarin use was associated with mortality and poor outcome in patients with SICH^34,35^, the impact of prior DOAC use on in-hospital mortality remains inconclusive^36,37^. A nationwide, cross-sectional Japanese analysis showed that prior use of DOAC (DOAC-ICH) was associated with significantly lower in-hospital mortality than prior use of warfarin (vitamin k antagonist (VKA)-ICH)) in SICH^38^. The rate of in-hospital mortality for DOAC-ICH was significantly lower than that for VKA-ICH at both 1 day and discharge (2.6% vs 6.5% at 1 day, and 17.6% vs 25.3% at discharge, respectively). Similar trends between DOAC-ICH and VKA-ICH were found in our results (1.4% vs 8.1% at 1 day and 11.3% vs 21.6% at discharge). Recent reports from Japan and Korea have also shown a lower rate of mortality in DOAC-ICH than in VKA-ICH^24,39^. These data may imply the greater safety of DOACs compared to warfarin for thromboembolic prevention in patients with a high risk of ICH. Compared to those studies from East Asia, higher rates of in-hospital mortality from SICH were reported both in DOAC-ICH (26.5–63.1%) and in VKA-ICH (32.6–62.7%) from the United States and European countries^26,27,40,41^. The reasons of different rates of in-hospital mortality are not known between different regions, but treatment strategies including surgery or early referral to stroke unit might influence the case fatality^2^. Although one meta-analysis showed that VKA-ICH was significantly associated with higher in-hospital mortality as well as larger hematoma volume, and enlargement of hematoma compared to cases of SICH with no anticoagulant^35^, no such relationship was noted between prior use of DOAC and no prior use of OAC due to an insufficient number of studies. Several meta-analyses have compared outcomes between VKA-ICH and DOAC-ICH, yielding inconclusive result^42–44^, but data assessments of outcomes among patients with prior AT use compared to control (no prior AT) have remained scarce. Only one single center analysis from Amsterdam reported the risk of mortality and poor outcome with prior use of VKA compared to no prior AT medication^27^. Our data showed that the mortality rate within 24 h from hospitalization was lower in no ATT and DOAC-ICH (1.5% and 1.4%) than in AP or VKA-ICH (5.8% and 8.1%). This might have affected the lower total mortality rate in DOAC-ICH. Much larger HV and greater neurological severity might be associate with hyperacute fatality among this group, but HV was small (18 ml) in a case with DOAC-ICH in this study. In patients who died after more than 24 h, HG might play an important role for mortality in addition to baseline HV and neurological severity, especially in cases with VKA-ICH. In this regard, a larger study comparing hematological characteristics between DOAC-ICH and VKA-ICH is warranted.

Use of a reversal agent may improve outcomes for patients with VKA-ICH ^45^, but only 24% of patients with VKA-ICH and PT-INR ≥ 2 used a reversal agent such as PCC or FFP in this study. We could not find significant differences in hematoma growth or in-hospital mortality between groups with and without use of a reversal agent (data not shown), but the lower total use of reversal agents among patients receiving warfarin might have affected the study outcomes. In DOAC-ICH, only four patients (5.6%) received a reversal agent (two with PCC, one with FFP, and one with idarucizumab, respectively). The reversal agent for factor Xa inhibitors (andexanet alfa) had not been available during the study periods in Japan. A recent meta-analysis showed the mortality rate of DOAC-related bleeding was higher in patients with intracranial bleeding than in those with extracranial bleeding, and effective hemostasis was significantly associated with lower risk of death^46^. The efficacy and safety of specific reversal agents to DOAC (idarucizumab and andexanet alfa) compared to a non-specific traditional reversal agent (FFP or PCC) remains inconclusive in DOAC-ICH^47^. Thus, further studies are warranted to determine the net clinical benefits of reversal agents and to clarify which reversal agents are most beneficial for DOAC-ICH.

Our results also showed that concomitant use of AP and warfarin was associated with much higher in-hospital mortality than warfarin alone after SICH, while no such association was observed among patients on DOACs. Dual antithrombotic therapy (DAT) with warfarin (warfarin plus single antiplatelet) has been shown to increase the risk of intracranial bleeding to three times that with warfarin alone in elderly AF patients^48^. One meta-analysis that compared safety and efficacy of either DAT with DOAC or with warfarin in patients with AF undergoing percutaneous coronary intervention (PCI) showed that the risk of major bleeding was significantly lower in DAT with DOAC than with warfarin (risk ratio 0.59, 95% CI 0.49–0.71), and without any increase in cardiovascular events^49^. Thus, DAT with DOACs could be beneficial to reduce the risk of SICH compared to DAT with warfarin. A nationwide study from the United States with a large number of cases showed that in-hospital mortality was significantly higher with prior use of both warfarin (aOR 1.62, 97.5% CI, 1.53–1.71) and DOAC (aOR 1.21, 97.5% CI 1.11–1.32) compared to no prior use of OAC in SICH, and DOAC-ICH showed a lower risk of in-hospital mortality than VKA-ICH (aOR 0.75, 97.5% CI 0.69–0.81)^26^. Concomitant use of an antiplatelet significantly increased the risk of in-hospital mortality in VKA-ICH compared to warfarin alone, but the same trend was not found among patients with DOAC-ICH^26^. A recent sub-analysis of the PASTA registry also demonstrated that prior use of the combination of warfarin and antiplatelet was significantly associated with in-hospital mortality compared to DOAC monotherapy (OR 20.57, 95% CI 1.75-241.75) in patients with SICH^39^. Because of the limited number of enrolled patients and lower statistical power, we could not confirm an independent association between DAT with warfarin and in-hospital morality compared to no prior AT use in this study. However, in-hospital mortality was about 7 times higher in VKA-ICH with antiplatelet compared to no prior AT use (37.5% vs 5.2%). The larger HV and higher rate of intraventricular hemorrhage in AP + Warfarin probably increased the risk of in-hospital mortality compared to warfarin alone, but no such effect was not found in groups with DOAC-ICH. We did not collect the time of last DOAC intake before ICH in this study. The association between blood concentration of DOAC and outcome remained unclear for DOAC-ICH. Cerebellar hemorrhage may also increase the risk of in-hospital mortality by compressing the brainstem or increasing the risk of hydrocephalus. The prevalence of cerebellar hemorrhage was highest in AP + warfarin (25.0%), but none of those patients had died by discharge in this study.

DAT with warfarin represents a significant risk for SICH and following high in-hospital mortality, as demonstrated by the current and previous studies. Thus, when physicians use ATs, monotherapy with DOAC should be considered favorably. If patients need to be treated by DAT, warfarin should not be the first choice. Usage of non-pharmacological devices such as left atrial appendage closure will also be beneficial in patients with AF who have to use DAT with warfarin to reduce the risk of SICH^50^.

Several limitations should be considered. First, this was a prospective study with some retrospective data using a multi-center registry, but not all patients were enrolled. These issues may have introduced selection biases. Second, the number of patients with prior use of OACs, particularly warfarin, was very small. However, a post hoc power calculation found relatively sufficient statistical power (81%) for in-hospital mortality among patients with prior use of warfarin compared to no prior AT use. Third, information on the timing of last intake of DOAC and administration of each reversal agent was unavailable, and might have influenced hematoma characteristics and outcomes. Fourth, patients receiving OACs were older, more frequently had comorbidities, and showed higher HV. These background and clinical conditions might have affected treatment decisions or the selection of aggressive treatments and subsequent care, which would have related to final outcomes. Fifth, compared to those receiving antiplatelets and DOACs, patients on warfarin might have experienced more severe comorbidities such as cardiac valvular disease, cardiac valve replacement, and several coexisting heart diseases requiring AP and warfarin. These issues were not investigated in this study and might have contributed to the prognosis in this study.

## Conclusion

Among patients with Japanese SICH, a different trend was seen in prior AT use compared to before DOACs became available. In patients with prior use of OACs, DOACs were used at almost double the rate of warfarin. Prior warfarin use, but not prior use of AP or DOACs, was a significant risk for in-hospital mortality compared to no prior AT use. DAT with warfarin may further increase the risk of in-hospital mortality after SICH.

### Supplementary Information

Supplementary Table 1.

Supplementary Table 2.

Supplementary Table 3.

Supplementary Table 4.

Supplementary Table 5.

## Data Availability

The datasets used and/or analyzed in the current study are available from the corresponding author on reasonable request.

## Abbreviations

AF: Atrial fibrillation
aOR: Adjusted odds ratio
AP: Antiplatelet
AT: Antithrombotic
ATT: Antithrombotic therapy
BMI: Body mass index
DAT: Dual antithrombotic therapy
DOAC: Direct oral anticoagulant
FFP: Fresh frozen plasma
HG: Hematoma growth
HV: Hematoma volume
ICH: Intracerebral hemorrhage
IVH: Intraventricular hemorrhage
MI: Myocardial infarction
mRS: Modified Rankin Scale
NIHSS: National Institutes of Health Stroke Scale
OAC: Oral anticoagulant
PCC: Prothrombin complex concentrate
PT-INR: Prothrombin time international normalized ratio
SICH: Spontaneous intracerebral hemorrhage
VKA: Vitamin K antagonist
