# Supplementary material for: Trends in prior antithrombotic medication and risk of in-hospital mortality after spontaneous intracerebral hemorrhage: the J-ICH registry
Source: Sci Rep. 2024 May 25;14:12009. doi: 10.1038/s41598-024-62717-5 (PMC11127931; doi:10.1038/s41598-024-62717-5)
Supplement: Supplementary file 1 — Supplementary Table 1. [file 41598_2024_62717_MOESM1_ESM.pdf]

Trends in prior antithrombotic medication and risk of in-hospital mortality after spontaneous intracerebral hemorrhage: the J-ICH registry

Hideaki Ueno [1]; Joji Tokugawa [2]; Rikizo Saito [3]; Kazuo Yamashiro [4]; Satoshi Tsutsumi [5]; Munetaka Yamamoto [6]; Yuji Ueno [7,8]; Makiko Mieno [9]; Takuji Yamamoto [1]; Makoto Hishii [2]; Yukimasa Yasumoto [5]; Chikashi Maruki [3]; Akihide Kondo [6]; Takao Urabe [4]; Nobutaka Hattori [8]; Hajime Arai [6]; and Ryota Tanaka [8,10*]

On behalf of the J-ICH Investigators

Supplemental table 1. Details of prior antiplatelet use in patients with SICH

**Prior antiplatelet use (All)**	**N=174**
Single antiplatelet therapy (SAPT)	N=148
Aspirin	102 (58.6%)
Clopidogrel	21 (12.1%)
Cilostazol	20 (11.5%)
Ticlopidine	3 (1.7%)
Prasugrel	2 (1.1%)
Dual antiplatelet therapy (DAPT)	N=26
Aspirin + Clopidogrel	14 (8.0%)
Aspirin + Prasugrel	5 (2.9%)
Aspirin + Cilostazol	4 (2.3%)
Clopidogrel + Cilostazol	2 (1.1%)
Aspirin + Ticlopidine	1 (0.6%)
