# Supplementary material for: Trends in prior antithrombotic medication and risk of in-hospital mortality after spontaneous intracerebral hemorrhage: the J-ICH registry
Source: Sci Rep. 2024 May 25;14:12009. doi: 10.1038/s41598-024-62717-5 (PMC11127931; doi:10.1038/s41598-024-62717-5)
Supplement: Supplementary file 2 — Supplementary Table 2. [file 41598_2024_62717_MOESM2_ESM.pdf]

Trends in prior antithrombotic medication and risk of in-hospital mortality after spontaneous intracerebral hemorrhage: the J-ICH registry

Hideaki Ueno [1]; Joji Tokugawa [2]; Rikizo Saito [3]; Kazuo Yamashiro [4]; Satoshi Tsutsumi [5]; Munetaka Yamamoto [6]; Yuji Ueno [7,8]; Makiko Mieno [9]; Takuji Yamamoto [1]; Makoto Hishii [2]; Yukimasa Yasumoto [5]; Chikashi Maruki [3]; Akihide Kondo [6]; Takao Urabe [4]; Nobutaka Hattori [8]; Hajime Arai [6]; and Ryota Tanaka [8,10*]

On behalf of the J-ICH Investigators

Supplemental table 2. Hematoma location by each age group (P<0.0001) and by with or without each antithrombotics (P<0.05)

	**Putamen**	**Thalamus**	**Lobes**	**Cerebellum**	**Brainstem**	**Caudate**	**Mixed/Others**
Total	365 (33.6%)	276 (25.4%)	254 (23.4%)	81 (7.5%)	61 (5.6%)	9 (0.8%)	39 (3.6%)
<55 years	98 (55.7%)	28 (15.9%)	19 (10.8%)	7 (4.0%)	16 (9.1%)	3 (1.7%)	5 (2.8%)
55–64 years	85 (42.7%)	53 (26.6%)	26 (13.1%)	15 (7.5%)	9 (4.5%)	4 (2.0%)	7 (3.5%)
65–74 years	85 (31.8%)	72 (27.0%)	60 (22.5%)	20 (7.5%)	15 (5.6%)	1 (0.4%)	14 (5.2%)
≥75 years	97 (21.9%)	123 (27.8%)	149 (33.6%)	39 (8.8%)	21 (4.7%)	1 (0.2%)	13 (2.9%)
No ATT	307 (37.3%)	196 (23.8%)	187 (22.7%)	53 (6.4%)	46 (5.6%)	9 (1.1%)	25 (3.0%)
AP	29 (18.8%)	50 (32.5%)	38 (24.7%)	16 (10.4%)	11 (7.1%)	0	10 (6.5%)
DOAC	17 (23.9%)	22 (31.0%)	23 (32.4%)	7 (9.9%)	1 (1.4%)	0	1 (1.4%)
Warfarin	12 (32.3%)	8 (21.6%)	6 (16.2%)	5 (13.5%)	3 (8.1%)	0	3 (8.1%)
