# Supplementary material for: Trends in prior antithrombotic medication and risk of in-hospital mortality after spontaneous intracerebral hemorrhage: the J-ICH registry
Source: Sci Rep. 2024 May 25;14:12009. doi: 10.1038/s41598-024-62717-5 (PMC11127931; doi:10.1038/s41598-024-62717-5)
Supplement: Supplementary file 3 — Supplementary Table 3. [file 41598_2024_62717_MOESM3_ESM.pdf]

Trends in prior antithrombotic medication and risk of in-hospital mortality after spontaneous intracerebral hemorrhage: the J-ICH registry

Hideaki Ueno [1]; Joji Tokugawa [2]; Rikizo Saito [3]; Kazuo Yamashiro [4]; Satoshi Tsutsumi [5];

Munetaka Yamamoto [6]; Yuji Ueno [7,8]; Makiko Mieno [9]; Takuji Yamamoto [1]; Makoto Hishii [2];

Yukimasa Yasumoto [5]; Chikashi Maruki [3]; Akihide Kondo [6]; Takao Urabe [4]; Nobutaka Hattori [8];

Hajime Arai [6]; and Ryota Tanaka [8,10*]

On behalf of the J-ICH Investigators

Supplemental table 3. Reversal agent use for Warfarin or DOAC

**Reversal agent**	**Warfarin (n=37)**	**DOAC (n=71)**
Vitamin K2 (menatetrenone)	23 (62.2%)	0
Prothrombin complex concentrate (PCC)	8 (21.6%)	2 (2.8%)
Fresh frozen plasma (FFP)	1 (2.7%)	1 (1.4%)
Idarucizumab	0	1 (1.4%)
