# Supplementary material for: Trends in prior antithrombotic medication and risk of in-hospital mortality after spontaneous intracerebral hemorrhage: the J-ICH registry
Source: Sci Rep. 2024 May 25;14:12009. doi: 10.1038/s41598-024-62717-5 (PMC11127931; doi:10.1038/s41598-024-62717-5)
Supplement: Supplementary file 4 — Supplementary Table 4. [file 41598_2024_62717_MOESM4_ESM.pdf]

Trends in prior antithrombotic medication and risk of in-hospital mortality after spontaneous intracerebral hemorrhage: the J-ICH registry

Hideaki Ueno [1]; Joji Tokugawa [2]; Rikizo Saito [3]; Kazuo Yamashiro [4]; Satoshi Tsutsumi [5]; Munetaka Yamamoto [6]; Yuji Ueno [7,8]; Makiko Mieno [9]; Takuji Yamamoto [1]; Makoto Hishii [2]; Yukimasa Yasumoto [5]; Chikashi Maruki [3]; Akihide Kondo [6]; Takao Urabe [4]; Nobutaka Hattori [8]; Hajime Arai [6]; and Ryota Tanaka [8,10*]

On behalf of the J-ICH Investigators

Supplemental table 4. Baseline characteristics and clinical outcomes according to types of DOAC

	Dabigatran (N=3)	Rivaroxaban (N=27)	Apixaban (N=21)	Edoxaban (N=20)	P value
Age, median (IQR), y	77 (75-80)	76 (60-83)	80 (68-84)	79 (73.5-84.8)	0.718
Sex (M), %	1 (33.3%)	18 (66.7%)	16 (76.2%)	13 (65.0%)	0.496
BMI, median (IQR), kg/m^2	21.6±2.9	23.7±4.1	23.1±4.8	22.0±3.2	0.682
Pre-stroke mRS≧3, (%)	0	3 (11.1%)	1 (4.8%)	1 (5.0%)	0.752
Vascular risks and medical history, No. (%)					
Hypertension	1 (33.3%)	17 (62.7%)	15 (71.4%)	15 (75.0%)	0.473
Dyslipidemia	2 (66.7%)	7 (25.9%)	8 (38.1%)	9 (45.0%)	0.373
Diabetes mellitus	0	7 (25.9%)	6 (28.6%)	4 (20.0%)	0.701
Atrial fibrillation	3 (100.0%)	21 (77.8%)	19 (90.5%)	15 (75.0%)	0.452
Coronary artery disease	0	8 (29.6%)	4 (19.1%)	4 (20.0%)	0.600

Ischemic stroke	0	4 (14.8%)	2 (9.5%)	5 (25.0%)	0.473
Hemorrhagic stroke	0	3 (11.1%)	2 (9.5%)	4 (20.0%)	0.648
Antiplatelet use, (%)	1 (33.3%)	4 (14.8%)	3 (12.3%)	4 (20.0%)	0.826
Acute status and treatment					
SBP at arrival, median (IQR) mmHg	169 (157-182)	170 (149-182)	168 (146.5-193.5)	165 (145-183.8)	0.882
NIHSS at arrival, median (IQR)	5 (1-24)	14 (5-26)	12 (3-17.5)	10.5 (4.5-15)	0.639
Hematoma location and characters					0.059
Supratentorial deep	0	14 (51.9%)	9 (42.9%)	16 (80.0%)	
Lobes	3 (100.0%)	8 (29.6%)	8 (38.1%)	4 (20.0%)	
Cerebellum/Brainstem	0	5 (18.5%)	3 (14.3%)	0	
Other	0	0	1 (4.8%)	0	
Hematoma volume, median (IQR), ml	33 (5.9-70)	22.6 (10-43)	18 (8.9-47.5)	8.4 (5.3-40)	0.311
Intraventricular expansion, (%)	0	11 (40.7%)	7 (33.3%)	10 (50.0%)	0.357
Hematoma growth, (%)	0	1 (3.7%)	3 (14.3%)	4 (20.0%)	0.303
Treatment					
Any surgery, (%)	1 (33.3%)	9 (33.3%)	4 (19.1%)	4 (20.0%)	0.625
Reversal agent use, (%)	1 (33.3%)	1 (3.7%)	2 (9.5%)	0	0.097
Outcomes, n (%)					
In-hospital mortality	0	3 (11.1%)	3 (14.3%)	2 (10.0%)	0.895
Hospital death within 24 hours	0	0	1 (4.8%)	0	0.491
Poor outcome (mRS5-6 at discharge)	0	8 (29.6%)	3 (14.3%)	5 (25.0%)	0.468
