# Supplementary material for: Trends in prior antithrombotic medication and risk of in-hospital mortality after spontaneous intracerebral hemorrhage: the J-ICH registry
Source: Sci Rep. 2024 May 25;14:12009. doi: 10.1038/s41598-024-62717-5 (PMC11127931; doi:10.1038/s41598-024-62717-5)
Supplement: Supplementary file 5 — Supplementary Table 5. [file 41598_2024_62717_MOESM5_ESM.pdf]

Trends in prior antithrombotic medication and risk of in-hospital mortality after spontaneous intracerebral hemorrhage: the J-ICH registry

Hideaki Ueno [1]; Joji Tokugawa [2]; Rikizo Saito [3]; Kazuo Yamashiro [4]; Satoshi Tsutsumi [5]; Munetaka Yamamoto [6]; Yuji Ueno [7,8]; Makiko Mieno [9]; Takuji Yamamoto [1]; Makoto Hishii [2]; Yukimasa Yasumoto [5]; Chikashi Maruki [3]; Akihide Kondo [6]; Takao Urabe [4]; Nobutaka Hattori [8]; Hajime Arai [6]; and Ryota Tanaka [8,10*]

On behalf of the J-ICH Investigators

Supplemental table 5. Univariate odds ratio for the risk of in-hospital mortality, hospital death within 24 h, and poor outcome (mRS 5-6 at discharge)

	**In-hospital mortality**			**Hospital death within 24h**			**Poor outcome (mRS 5-6 at discharge)**		
	OR	95%CI	P value	OR	95%CI	P value	OR	95%CI	P value
Age, median (IQR), y	1.02	1.00-1.04	0.0225	1.01	0.98-1.04	0.5670	1.03	1.02-1.04	<0.0001
Sex (male), n (%)	1.39	0.87-2.22	0.1649	2.13	0.88-5.14	0.0931	0.89	0.65-1.21	0.4527
BMI, kg/m^2	1	0.95-1.04	0.8558	1.02	0.95-1.09	0.5656	0.98	0.95-1.01	0.1755
Pre-stroke mRS ≥3, n (%)	1.75	0.81-1.24	0.1573	0.62	0.08-4.63	0.6393	2.41	1.42-4.08	0.0019
Current smoker, n (%)	0.66	0.35-1.3	0.24	0.59	0.17-1.98	0.3896	0.59	0.38-0.92	0.0159
Regular drinker, n (%)	0.43	0.22-0.82	0.011	0.24	0.06-1.03	0.0547	0.38	0.25-0.59	<0.0001
Vascular risks and medical history, n (%)									
Hypertension	0.64	0.40-1.01	0.061	0.78	0.34-1.77	0.5472	0.76	0.55-1.05	0.101
Dyslipidemia	0.83	0.51-1.36	0.4628	1.47	0.66-3.27	0.3439	1.36	0.99-1.85	0.057
Diabetes mellitus	1.64	0.97-2.77	0.0633	1.75	0.72-4.24	0.2176	1.8	1.25-2.57	0.0015
Atrial fibrillation	1.39	0.67-2.88	0.3759	1.47	0.43-5.00	0.538	1.32	0.79-2.20	0.2937

Hemodialysis	5.94	2.90-12.16	<0.0001	5.27	1.72-16.12	0.0036	3.68	1.95-6.96	<0.0001
Coronary artery disease	3.44	1.75-6.78	0.0003	3.35	1.11-10.09	0.0315	2.46	1.42-4.28	0.0014
Ischemic stroke	1.71	0.89-3.27	0.1053	3.03	1.18-7.77	0.0208	1.77	1.12-2.79	0.0148
Hemorrhagic stroke	0.47	0.17-1.30	0.1446	4.74	0	0.9867	1.1	0.66-1.82	0.7198
Acute status, hematoma characters, and treatment									
SBP at arrival, median (IQR), mmHg	1.01	1.0-1.01	0.0634	1.02	1.00-1.03	0.0014	1.01	1.00-1.01	0.0173
NIHSS on arrival, median (IQR)	1.15	1.12-1.18	<0.0001	1.17	1.12-1.22	<0.0001	1.14	1.12-1.17	<0.0001
Hematoma volume, ml	1.03	1.02-1.03	<0.0001	1.02	1.01-1.03	<0.0001	1.03	1.03-1.04	<0.0001
Intraventricular expansion, (%)	7.58	4.37-13.16	<0.0001	13.62	4.05-45.80	<0.0001	5.56	3.99-7.76	<0.0001
Hematoma growth, (%)	4.55	2.21-9.38	<0.0001	1.37	0	0.9867	3.8	2.06-6.98	<0.0001
Any surgery, (%)	1.05	0.62-1.76	0.8624	0.25	0.06-1.06	0.0608	2.81	2.03-3.87	<0.0001
Reversal agent use (FFP, PCC, Idar)	1.05	0.13-8.16	0.9647	3.85	0	0.9891	3.85	1.28-11.60	0.0163
